# Editorial: Neonatal ECMO in 2019: Where Are We Now? Where Next?

**DOI:** 10.3389/fped.2021.796670

**Published:** 2022-01-04

**Authors:** Giacomo Cavallaro, Matteo Di Nardo, Aparna Hoskote, Dick Tibboel

**Affiliations:** ^1^Neonatal Intensive Care Unit, Fondazione IRCCS Ca' Granda Ospedale Maggiore Policlinico, Milan, Italy; ^2^Pediatric Intensive Care Unit, Children's Hospital Bambino Gesù, IRCCS, Rome, Italy; ^3^Cardiorespiratory and Critical Care Division, Great Ormond Street Hospital for Children NHS Foundation Trust, London, United Kingdom; ^4^Intensive Care and Department of Pediatric Surgery, Erasmus MC-Sophia Children's Hospital, Rotterdam, Netherlands

**Keywords:** newborn, developmental hemostasis, extracorporeal circulation, cardiac ECMO, sepsis, congenital diaphragmatic hernia, meconium aspiration syndrome, pharmacology

Despite significant advances in neonatal intensive care, including neonatal ventilation in the current era, extracorporeal membrane oxygenation (ECMO) continues to play a crucial role in selected cases of severe cardio-respiratory failure, potentially reversible, but refractory to conventional ventilatory therapy and maximal pharmacological treatment (1).

Our Research Topic attempted to focus on some of continuing challenges in neonatal ECMO. In this issue of Frontiers in Pediatrics, we have collected a wide range of manuscripts related to the use of ECMO in the neonatal period (Broman; Butt and Chiletti; Cashen et al.; Di Nardo et al.; Kersten et al.; Macchini et al.; Perez Ortiz et al.; Rafat and Schaible; Raffaeli et al.; Raffaeli et al.; Roeleveld and Mendonca; Schiller and Tibboel).

Since the formation of the Extracorporeal Life Support Organization (ELSO) in 1989, 45,205 newborns have been supported on ECMO in 492 centers (www.elso.org) (2). Respiratory failure was the predominant reason for ECMO utilization in 33,400 newborns, whereas ECMO was used for cardiac failure in 9,561 newborns, and 2,244 were supported for refractory cardiac arrest—extracorporeal cardiopulmonary resuscitation (ECPR). Today, congenital diaphragmatic hernia (CDH) and meconium aspiration syndrome (MAS) are the exclusive neonatal diagnoses that alone represent about 46% of all cases of neonatal respiratory ECMO, reaching 92% of total ECMO if all “others” neonatal ECMO were added (2, 3). The classification of “others” includes all other diagnostic categories such as non-specific respiratory failure, congenital anomaly, pulmonary hypoplasia, hypoxic-ischemic encephalopathy, cardiorespiratory arrest, and inborn errors of metabolism (4). The mortality rate, however, varies significantly depending on the underlying respiratory disease. For instance, neonates with CDH and sepsis have higher mortality rates (47 and 49%, respectively) in contrast to those with MAS (9%) (2). Pulmonary hypertension and lung hypoplasia play a crucial role in determining survival in CDH (5). Neonates with prolonged ECMO run for >21 days have demonstrated higher mortality due to the increased risk of mechanical complications (6).

Veno-arterial (V-A) ECMO still represents the support of choice in neonates, with more than 80% receiving V-A support (2). The vessel size is the most critical limiting factor in using the veno-venous (V-V) ECMO in neonates as the smallest double-lumen venous cannula currently commercially available is 13 Fr (3, 7). However, it should be noted that mortality is not significantly different between the two types of support. However, neurological complications are reported to be lower in V-V support as compared to V-A support, although factors other than just the cannulation may account for this (3, 8).

With a wider spectrum of indications for ECMO utilization in the neonatal period as evidenced by the “others” diagnostic category in the ELSO Registry, we speculate that there is greater use of ECMO as compared to a few decades earlier (2).

Several unanswered questions remain on the use of ECMO in CDH [Rafat and Schaible; (9–11)]. The survival is dependent on several factors such as the side and size of the defect, pulmonary hypertension, associated abnormalities, gestational age at birth, and treatment (12–14). The prenatal and postnatal factors that are predictive of mortality, pulmonary hypertension, and the need for ECMO are the focus of many research groups (15). While there are scores developed from the ELSO Registry to predict outcome from ECMO in CDH, these are not to be factored in for patient selection which has to be individualized per patient. An alternative approach using the machine learning approach of the different variables that affect mortality may contribute to developing a reliable and safe predictive model (16).

Until recently, surgical procedures (excluding cardiac surgery and CDH repair) on ECMO remain infrequent (17). Bleeding has been the most feared major complication, although there was no associated increased incidence of mortality (17). Kersten et al. reported the neonatal and pediatric outcomes of surgery on ECMO (other than CDH repair), noting that 14% of patients in their series required surgery, of whom 50% had a poor prognosis. For neonates with congenital tracheobronchial malformations surgery, surgery on ECMO would have the advantage of lower anticoagulation and a wider operating field than CPB. In addition, postoperative ECMO would allow a period of lung rest better than conventional ventilation alone (3).

While pneumonia and neonatal sepsis remain an indication for ECMO support, the use of ECMO in this context has decreased like other neonatal indications. Furthermore, ECMO did not modify the high incidence of mortality related to neonatal septic shock (18), but there are some conflicting data, with some studies reporting 77% survival and others reporting 25% survival (18–20).

The ELSO indications for ECMO have remained unchanged for infants in whom sepsis is associated with pulmonary hypertension, right ventricular dysfunction, and hypoxemia (21). For those in whom sepsis presents with systemic inflammatory response, refractory septic shock, and multi-organ failure, the only indication for ECMO is treatment-resistant hypotension (21). However, time to initiation, mode of ECMO (V-V vs. V-A ECMO), ECMO flow rates, and run length remain controversial (20, 22). Therefore, the International Guidelines for the Management of Septic Shock in Children are weak evidence for recommendation on using V-V ECMO in children with sepsis-induced pediatric acute respiratory distress syndrome and refractory hypoxia. Similarly, the advice concerning V-A ECMO as a rescue treatment in children with septic shock refractory to all other therapies is weak (23).

Bleeding and thrombosis continue to be the most common complications during neonatal ECMO and are associated with increased morbidity and mortality (2, 24, 25). Knowledge of developmental hemostasis, and accurate titrated use of unfractionated heparin (UFH), with the integration of point-of-care monitoring systems based on whole blood [activated clot time (ACT), thromboelastography (TEG), or thromboelastometry (ROTEM)] to plasma tests [activated partial thromboplastin time (APTT) and anti-Factor Xa], may reduce hemorrhagic and thrombotic side effects during neonatal ECMO [Cashen et al.; Perez Ortiz et al.; (26, 27)].

In recent years, single-center studies with limited patient numbers have been published on the use of thrombin inhibitors (bivalirudin, argatroban, lepirudin) (28, 29). These thrombin inhibitors directly inhibit both bound and free thrombin and are antithrombin independent (30). However, their half-life is relatively long compared to UFH (28, 31). These safety and dosing concerns and lack of reversibility make direct thrombin inhibitors less attractive in the neonatal ECMO population as a first-line agent.

Although the indications and cases of neonatal respiratory ECMO decreased, number of cardiac ECMO cases has progressively increased, even though survival remained low ~40% (2). The indications for cardiac ECMO include pre-operative hemodynamic stabilization, failure of weaning from cardiopulmonary bypass, low cardiac output syndrome after cardiac surgery, and ECPR (32, 33). The incidence of postoperative ECMO currently varies from center to center and ranges from 1.4 to 5% (34). Any residual lesions should be promptly identified, and interventions should be immediately undertaken (35–37). The implementation of technical performance score as a predictor of early postoperative morbidity and early diagnosis with echocardiography and cardiac catheterization in the first 24 h after surgery is crucial to improve outcomes and survival (35, 36, 38–41).

During ECMO, drug pharmacokinetics (PK) and pharmacodynamics (PD) are modified by several factors related to the patient, drugs, circuits, and interactions (Raffaeli et al.). In addition, in newborns, maturational and non-maturational factors play a crucial role in PK and PD variability (Raffaeli et al.). However, the extensive PK variability during ECMO does not facilitate an adequate understanding of the developmental aspects of PD. A mathematical approach with Monte Carlo simulation or physiologically based pharmacokinetics (PBPK) could help these cases (Raffaeli et al.). Physiologically based pharmacokinetics is a knowledge-driven technique acquired in other settings, like other populations (adult, pediatric, neonatal), other drugs, or other sources (as *in vitro, in vivo, in silico* experiments), applying mathematical modeling for automatic integration (Raffaeli et al.). Furthermore, the development of virtual organs allows us to add variables to the model, to study any modification in terms of absorption, volume of distribution, and clearance according to the different ages, diseases, or extracorporeal supports (42).

Although the number of neonatal ECMO is constant, the centralization of ECMO delivery—the hub and spoke model—also throughout by ECMO transport service is needed, allowing continuous updating and improvement of knowledge through structured training programs, cost reduction, optimization of human and material resources, and improvement of assistance with a decrease of mortality and morbidity [Broman; Macchini et al.; (43–46)]. However, data in the literature are conflicting as some small programs have published excellent results with low mortality while some high-volume centers appear to have higher mortality that still seems to be linked to the patients' greater complexity (45, 47, 48). Moreover, keeping high quality in small ECMO programs presupposes an increase in training cost, ensuring a continuous training program, especially in machine troubleshooting and patient complications (43, 49, 50).

Independently to ECMO, follow-up of newborns with complex respiratory and cardiac pathologies is required to prevent and treat potential associated neurocognitive deficits. Therefore, long-term and multidisciplinary follow-up associated with neurorehabilitation strategies, as Cogmed working memory training, psychoeducation, compensatory techniques, and external aids, would appear to improve the lives of these tiny patients [ Schiller and Tibboel; (51)].

Even though ECMO was introduced several decades ago, it is still required for some clinical conditions that endanger the life of newborns. Therefore, its use must also be based on scientific evidence that deserves careful ethical consideration (Di Nardo et al.). The ethical question is no less critical than the indications of neonatal ECMO. Commonly, the family perceives the difference between rejection and withdrawal differently. In fact, complications during ECMO often would not justify the withdrawal of support in parents' eyes, while refusal to ECMO appears justified by contraindications (Di Nardo et al.).

Although much has been done to date, much more can be done by focusing on the points still open and, above all, by formalizing the research agenda among a network of hub centers that can work together, sharing successes and failures to improve the quality of care and life of these complex newborns. The futuristic concept of using the extracorporeal circulation of the extra-uterine environment for newborn development (EXTEND) program seems attractive to improve morbidity and mortality of extremely premature babies (23–25 weeks). The goal is to mimic a typical uterine environment and provide physiological support to the fetus (52–54). Thus, we could imagine our NICUs no longer full of incubators and pulmonary ventilators but rather full of wombs and artificial placentas where newborns develop while maintaining the normal physiological process.

Therefore, although we traveled a long road, we still have many more miles in front of us.

## Author Contributions

GC, MD, AH, and DT contributed to the study's conception and design. GC wrote the first draft of the manuscript. MD, AH, and DT provided extensive critical revision. All authors contributed to the manuscript's critical revision, read and approved the submitted version.

## Conflict of Interest

The authors declare that the research was conducted in the absence of any commercial or financial relationships that could be construed as a potential conflict of interest.

## Publisher's Note

All claims expressed in this article are solely those of the authors and do not necessarily represent those of their affiliated organizations, or those of the publisher, the editors and the reviewers. Any product that may be evaluated in this article, or claim that may be made by its manufacturer, is not guaranteed or endorsed by the publisher.
